# The Plastidial DIG5 Protein Affects Lateral Root Development by Regulating Flavonoid Biosynthesis and Auxin Transport in Arabidopsis

**DOI:** 10.3390/ijms231810642

**Published:** 2022-09-13

**Authors:** Wei Liu, Tao Chen, Yajie Liu, Quang Tri Le, Ruigang Wang, Hojoung Lee, Liming Xiong

**Affiliations:** 1 Donald Danforth Plant Science Center, St. Louis, MO 63132, USA; 2 High-Tech Research Center, Shandong Academy of Agricultural Sciences, Jinan 250100, China; 3 College of Life and Environmental Sciences, Hangzhou Normal University, Hangzhou 311121, China; 4 Department of Biology, Hong Kong Baptist University, Kowloon Tang, Hong Kong, China; 5 Department of Plant Biotechnology, College of Life Sciences and Biotechnology, Korea University, Seoul 136-713, Korea; 6 Inner Mongolia Key Laboratory of Plant Stress Physiology and Molecular Biology, Inner Mongolia Agricultural University, Hohhot 010010, China; 7 State Key Laboratory for Agribiotechnology, Chinese University of Hong Kong, Hong Kong, China

**Keywords:** lateral roots, root system architecture, flavonoid, polar auxin transport, chloroplast, retrograde signaling, tRNA adenosine deaminase arginine

## Abstract

To reveal the mechanisms underlying root adaptation to drought stress, we isolated and characterized an Arabidopsis mutant, *dig5* (*d*rought *i*nhibition of lateral root *g*rowth *5*), which exhibited increased sensitivity to the phytohormone abscisic acid (ABA) for the inhibition of lateral root growth. The *dig5* mutant also had fewer lateral roots under normal conditions and the aerial parts were yellowish with a lower level of chlorophylls. The mutant seedlings also displayed phenotypes indicative of impaired auxin transport, such as abnormal root curling, leaf venation defects, absence of apical hook formation, and reduced hypocotyl elongation in darkness. Auxin transport assays with [^3^H]-labeled indole acetic acid (IAA) confirmed that *dig5* roots were impaired in polar auxin transport. Map-based cloning and complementation assays indicated that the *DIG5* locus encodes a chloroplast-localized tRNA adenosine deaminase arginine (TADA) that is involved in chloroplast protein translation. The levels of flavonoids, which are naturally occurring auxin transport inhibitors in plants, were significantly higher in *dig5* roots than in the wild type roots. Further investigation showed that flavonoid biosynthetic genes were upregulated in *dig5*. Introduction of the flavonoid biosynthetic mutation *transparent testa 4* (*tt4)* into *dig5* restored the lateral root growth of *dig5*. Our study uncovers an important role of DIG5/TADA in retrogradely controlling flavonoid biosynthesis and lateral root development. We suggest that the DIG5-related signaling pathways, triggered likely by drought-induced chlorophyll breakdown and leaf senescence, may potentially help the plants to adapt to drought stress through optimizing the root system architecture.

## 1. Introduction

The root system physically anchors a plant in soil and is responsible for acquiring water and nutrients to support plant growth. Roots can also sense the soil environment to adjust their own growth and physiology and are able to communicate the information to the above ground parts to initiate appropriate responses in the whole plant. The plant root system is thus highly versatile and its coordination with the shoot is critical for the entire plant to adapt to adverse conditions, such as soil water deficit (drought). 

Drought is a common abiotic stress that plants encounter frequently. Even on well-irrigated agricultural land, periodic drought can occur on a daily basis. Upon encountering drought stress, plants can deploy both rapid and slow responses to deal with the stress. Rapid responses include closing stomata to minimize transpiration and activating a particular group of genes that are not expressed or expressed at low levels under normal conditions. The products of these stress-responsive genes may have diverse functions, such as facilitating osmotic adjustment and mitigating damages caused by stress. Slow responses are mostly related to developmental changes, such as altering stomata density, reinforcing leaf cuticles and waxes, promoting leaf senescence, and remodeling the root system [1,2,3,4]. The coordination of these various aspects of the drought stress response involves complex signal perception, transduction, and execution processes [3,5,6,7,8]. Understanding these signaling mechanisms is important for breeding crop tolerance to drought stress. Despite extensive research effort, many of the mechanisms underlying these diverse responses are still unclear. Major challenges, such as the complexity of drought stress as a signal, the difficulty in quantitatively and reproducibly applying drought treatments, and the lack of drought-specific responses in plants, have limited genetic studies of drought stress tolerance [5].

Root development has long been perceived as critical to plant drought stress tolerance, yet the particular traits of roots responsible for drought tolerance are rather obscure. Studies with model plants and analyses of quantitative trait loci (QTL) or genome-wide association studies (GWAS) in crop and other plants suggested that certain root traits may correlate with aspects of plant drought stress tolerance. These root traits include, for example, root biomass, diameter, length, surface area, root growth angle, presence of root hairs, and other properties, such as hydrotropism, anatomy, and hydraulic conductivity [9,10,11,12,13,14,15,16,17,18]. Yet many of these correlative relations lack strong genetic or other evidence to support their causal relationship. Furthermore, there are significant variations in the contributions of these traits to drought tolerance among different plant species.

While monocotyledonous plants have a fibrous root system, dicotyledonous plants such as *Arabidopsis thaliana* have a taproot system consisting of a main root (primary root) and multiple lateral roots growing out of the main root. Lateral roots develop from the pericycle cells adjacent to the xylem poles [19,20,21]. The development and growth of lateral roots are under the coordinated control of plant hormones, particularly auxin, and are strongly influenced by external conditions [21,22,23]. As a result, lateral root development is highly plastic and responsive to environmental conditions such as nutrient availability, soil mechanical property, and soil water status. Soil water deficit can dramatically affect root system architecture, including the development of lateral roots. Studies with Arabidopsis have indicated that osmotic stress and the phytohormone abscisic acid (ABA) can inhibit lateral root development [24,25,26]. We previously hypothesized that this response may be potentially linked to plant adaptation to drought and may underlie novel mechanisms of drought stress tolerance. We thus conducted genetic screens for *dig* (*d*rought *i*nhibition of lateral root *g*rowth) mutants that showed altered lateral root development in response to mannitol or ABA. Our study showed that there is a close link between this root response to drought or ABA and the whole plant drought tolerance [25]. We reasoned that reduced lateral root growth under drought stress may allow the plants to allocate more resources to grow deeper roots for water uptake, since water is often more available in deep soil [25]. Thus, the analysis of the lateral root response to drought may reveal novel determinants of plant drought tolerance. 

In this study, we characterized a mutant isolated in our genetic screen, named *dig5.* The *dig5* mutant plants showed increased inhibition by ABA of lateral root growth, although the mutant also had reduced lateral root growth under normal conditions. The *dig5* mutant plants were yellowish with a reduced growth rate and a short stature. The mutant exhibited various phenotypes indicative of impaired auxin transport, which was confirmed by auxin transport assays. Further investigations showed that *dig5* roots accumulated higher levels of flavonoids, secondary metabolites known to inhibit auxin transport. Introduction of the flavonoid biosynthesis mutation *tt4* into *dig5* restored lateral root growth of *dig5*. Map-based cloning found that the *DIG5* locus encodes a chloroplast tRNA adenosine deaminase arginine (TADA) that edits the Arg (ACG) wobble position of tRNA^Arg^ to inosine (ICG). Our study discovered an important role of a plastidial protein in controlling lateral root development through regulating flavonoid synthesis and polar auxin transport. We suggest that the DIG5-related pathways may help plants to adapt to drought conditions.

## 2. Results

### 2.1. Isolation and Characterization of the dig5 Mutant

To identify genes important for drought tolerance, we screened for mutants defective in lateral root responses to osmotic stress or ABA. Seeds of *Arabidopsis thaliana* Col-*gl1* (referred to as the wild type hereafter) were mutagenized and M_2_ seedlings were screened for mutants that showed more or less lateral root growth than the wild type under ABA or mannitol treatments. These mutants were referred to as *dig* (*d*rought *i*nhibition of *g*rowth of lateral roots) mutants [25] and one of these mutants, *dig5*, was characterized in this study.

**Figure 1:**
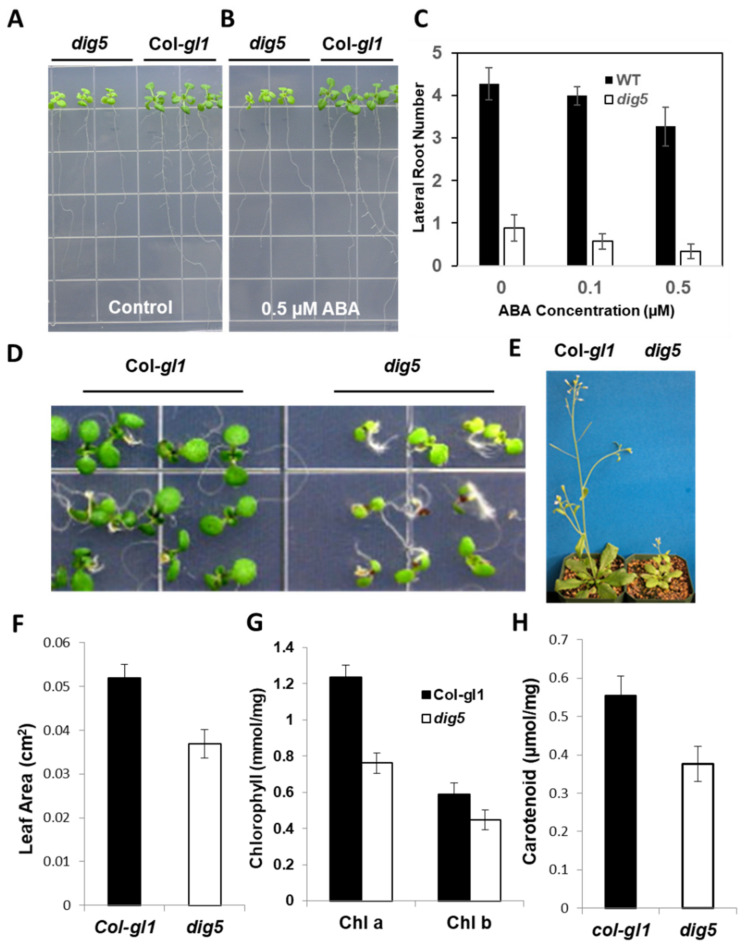
Phenotypes of the *dig5* mutant. (**A**–**C**) Response of lateral roots of the wild-type Col-*gl1* and *dig5* seedlings to ABA. Three-day-old seedlings were transferred to the shown plate (**A**) without (control) or (**B**) with 0.5 μM ABA and grown for 3 days before taking the pictures. (**C**) Lateral root number per seedling of the wild type and *dig5* shown in (**A**). Data are means ± SD from 8 to 9 seedlings. (**D**) Morphology of 7-day-old seedlings of the wild type and *dig5* growing on agar plates. (**E**) Morphology of adult plants growing in soil for one and a half months. (**F**) Leaf area of the first two rosette leaves of 1-week-old wild type and *dig5* mutants grown in agar media. (**G**) Chlorophyll contents of the wild type and *dig5* mutant leaves of 3-week-old seedlings grown in soil. (**H**) Carotenoid content of leaves of 3-week-old wild type and *dig5* seedlings grown in soil.

In the initial screen on 1/2 MS medium supplemented with 0.5 μM ABA, the wild type seedlings could still produce a reduced number of lateral roots compared with the control (without ABA), but the *dig5* mutant had nearly no visible lateral roots. In subsequent re-screens, however, the number of lateral roots of *dig5* was found to be fewer even on the control medium without ABA compared to wild type (Figure 1A). In media without ABA, the average number of visible lateral roots per plant in the wild type was 6 times that of *dig5* (Supplementary Table S1). In the presence of ABA, while the lateral root number and length of both the wild type and *dig5* decreased, these were decreased more in *dig5*, reflecting increased sensitivity to ABA inhibition of lateral root growth in the mutant (Figure 1A–C). On full-strength MS media (where lateral root development was suppressed compared with on 1/2 MS media), the primary root growth was inhibited more in *dig5* than in the wild type by ABA or NaCl (Supplementary Figure S1A–C), suggesting a general increase in ABA sensitivity in the *dig5* mutant. 

In addition to their reduced lateral root numbers, *dig5* mutant seedlings had other phenotypes distinct from the wild type. The primary root of the mutant was shorter, at 60 percent that of the wild type (Figure 1A and Supplementary Table S1). The seedlings of *dig5* were smaller in agar media (Figure 1D) and had a shorter stature when grown in soil (Figure 1E, Supplementary Table S1), which did not allow us to accurately compare their drought tolerance with the wild type due to the difference in their size. Another notable phenotype of the mutant was that the leaves were yellowish or pale green, either in MS media or in soil (Figure 1D,E). The contents of chlorophyll a, b, and carotenoids in *dig5* were significantly lower than in the wild type (Figure 1G,H). Detailed morphometric analyses showed that there were significant differences in seedlings and in mature plants between the *dig5* mutant and the wild type (Supplementary Table S1). For 8-day-old seedlings, the number of lateral roots in *dig5* was about 83.5% fewer than that of the wild type. For adult plants growing in soil, the inflorescence stem length of mutant plants was only 28.6% that of the wild type, and the silique number and distance between siliques were 26.8% and 53.5% those of the wild type, respectively (Supplementary Table S1). 

### 2.2. Auxin Responses of the dig5 Mutant

**Figure 2:**
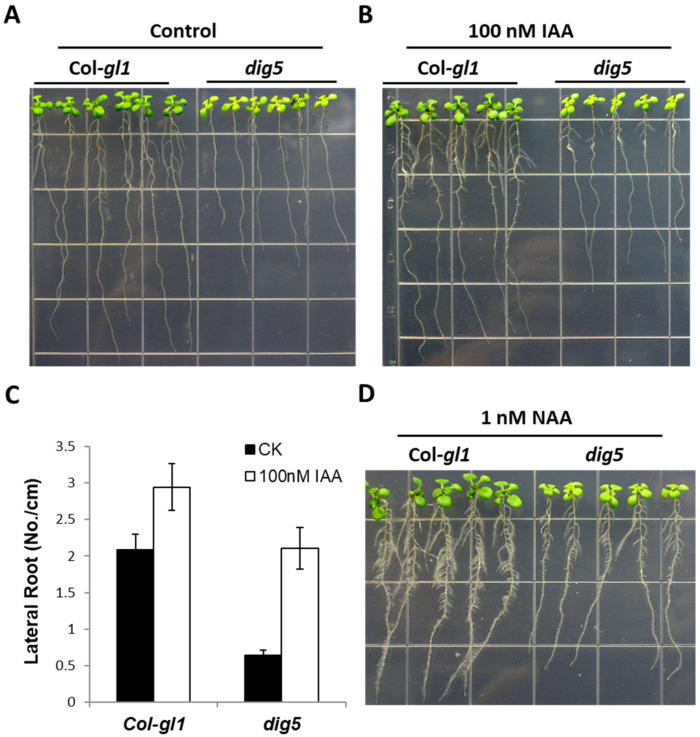
Lateral root phenotypes of *dig5* mutant seedlings can be rescued by auxin. Lateral root growth of the wild type (Col-*gl1*) and *dig5* without (**A**) or with (**B**) 100 nM IAA. (**C**) Lateral root density of seedlings shown in (**A**,**B**). Data are means and standard deviation (*n* = 18). (**D**) Morphology of seedlings of the wild type (Col-*gl1*) and *dig5* on 1 nM NAA.

The reduced number of lateral roots in the *dig5* mutant is striking. Since the phytohormone auxin plays critical roles in lateral root development [21], we examined whether the mutants were altered in auxin response. When growing in media containing the auxin indole-3-acetic acid (IAA), the number of lateral roots in both the wild type and *dig5* increased (Figure 2A,B). Although the total number of lateral roots in *dig5* was still lower than in the wild type, the increase in *dig5* was about 165% higher than the increase in the wild type (Figure 2C). Similarly, the synthetic auxin analogs 1-naphthaleneacetic acid (NAA) and 2,4-dichlorophenoxyacetic acid (2,4-D) also significantly increased the number of lateral roots in *dig5* (Figure 2D). These data indicated that *dig5* was responsive to auxin and that exogenous auxin could partly rescue the defects in lateral root development in *dig5.*

**Figure 3:**
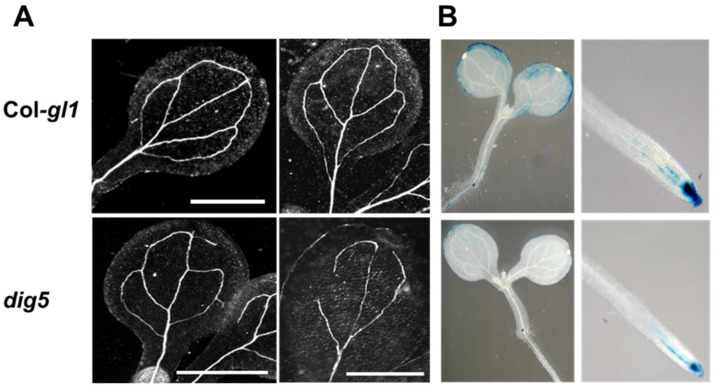
Leaf venation defects and reduced auxin levels in *dig5* mutant seedlings. (**A**) Representative pictures of leaf venation in seedlings of wild type (Col-*gl1*) and *dig5* mutant. Scale bars = 1 cm. (**B**) Expression of *DR5-GUS* reporter gene (GUS-staining) in wild type (Col-*gl1*) and *dig5* seedlings.

Auxins are also important in regulating the development of vasculature [27]. In contrast to the regular venation in the wild type leaves, *dig5* leaves often had irregular, disrupted, and discontinuous veins (Figure 3A). These phenotypes suggested that *dig5* mutants may have reduced levels of auxin or an impaired response to auxin. To test these possibilities, we introduced the auxin reporter gene *DR5-GUS* into the *dig5* mutant through genetic crossing. Young seedlings of the resulting lines were stained for GUS expression (Figure 3B). The *DR5-GUS* expression was evident in leaves and roots of the wild type seedling, particularly at the root tip, in vascular tissues of the root, and the edge of leaves. In contrast, the *DR5-GUS* signal was significantly weaker in *dig5* in both leaves and roots (Figure 3B). The nearly normal auxin response of the *dig5* mutant in the lateral root development and the reduced auxin levels in the root suggest that the *dig5* mutant may have a reduced auxin transport capability. We thus examined whether *dig5* was defective in other processes that are affected by auxin transport.

### 2.3. Altered Root Curling, Hypocotyl Elongation and Apical Hook Formation in dig5 

**Figure 4:**
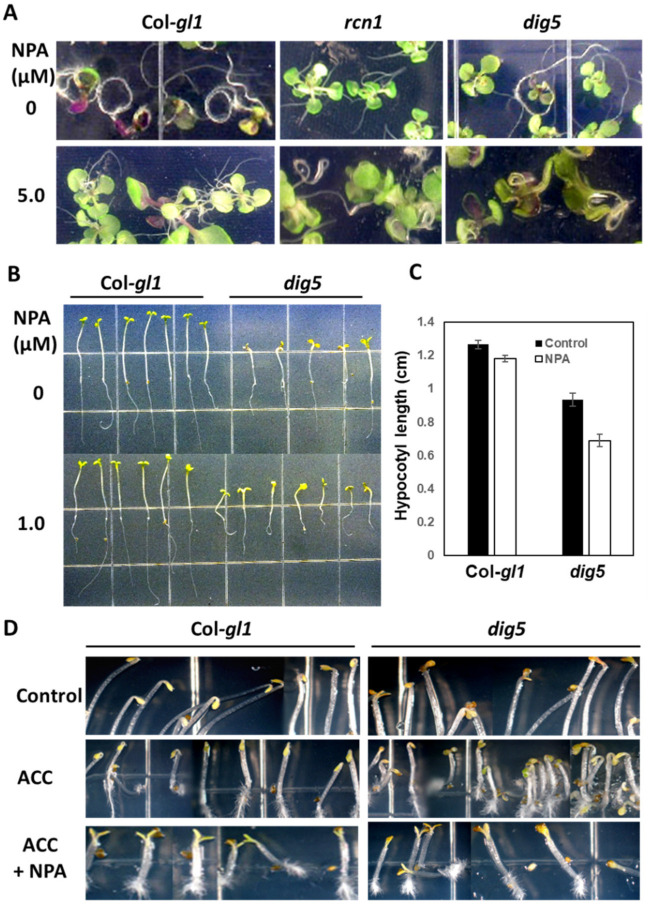
Abnormal root curling and apical hook formation of *dig5* mutants. (**A**) Root curling of Col-*gl1*, *rcn1*, and *dig5* seedlings in the absence or presence of 5 µM NPA. (B) Hypocotyl elongation of Col-*gl1* and *dig5* in the absence or presence of 1.0 µM NPA. (**C**) Hypocotyl length of seedlings in (**B**). Data are means and standard deviation from 18 seedlings. (**D**) Apical hook formation of seedlings under the control, ACC (10 µM), and ACC (10 µM) + NPA (1 µM) treatments. Seedlings were germinated and grown in darkness for 3 days before taking the pictures.

One of the phenotypes related to polar auxin transport in roots is root curling [28]. This phenotype can be seen with seedlings growing in a petri dish with agar media. When growing to the bottom of the agar plate, roots of the wild type seedlings will begin to curl in a circle-like pattern, presumably searching for the gravity vector. However, when the auxin transport inhibitor N-1-naphthylphthalamic acid (NPA) is added to the medium, roots grow in a straight line instead of curling. The auxin transport mutant *roots curl in NPA 1 (rcn1)* has an opposite phenotype to the wild type in that the roots do not curl in the absence of NPA but do curl strongly in the presence of NPA [28]. We examined the root curling behavior of *dig5* seedlings and included the *rcn1* mutant in the assay for comparison. In the control medium without NPA, the wild type roots curled as expected, but the *dig5* mutant roots grew straight without or only slightly curling. In the presence of NPA, the wild type roots grew straight, but the *dig5* roots curled (Figure 4A). Thus, the *dig5* mutant was defective in the root curling response to NPA and behaved like the *rcn1* mutant, consistent with the notion that *dig5* may be defective in polar auxin transport. 

Inhibition of auxin transport by NPA also inhibits hypocotyl elongation of dark-grown seedlings. We measured the sensitivity of hypocotyl elongation to NPA in the *dig5* mutant. Without NPA, *dig5* seedlings had shorter hypocotyls than the wild type. NPA treatment inhibited the hypocotyl elongation of both wild type and *dig5* seedlings, with the mutant hypocotyl inhibited by 27.3%, but the wild type by only 6.7% when compared with control treatments without NPA (Figure 4B,C).

Dark-grown Arabidopsis seedlings form apical hooks, and the hooks can be exaggerated by treating with ethylene or its precursors and can be reduced by treating with NPA. These responses are also mediated by auxin partly through auxin transport [28,29,30]. We examined apical hook formation in *dig5* mutant seedlings. The wild type seedlings showed tight apical hooks on normal MS media, as expected, and exaggerated hooks were seen with addition of the ethylene precursor 1-aminocyclopropane-1-carboxylate (ACC) (Figure 4D). In contrast, the *dig5* mutant seedlings grown under the same conditions did not form apical hooks or formed only partial hooks (Figure 4D). ACC also had limited enhancement on apical hook formation in *dig5* seedlings (Figure 4D). As a control, hook formation in the wild type as well as in *dig5* was blocked by NPA even in the presence of ACC (Figure 4D), consistent with the notion that polar auxin transport plays a dominant role in the apical hook formation. 

### 2.4. Polar Auxin Transport Was Impaired in dig5 

**Figure 5:**
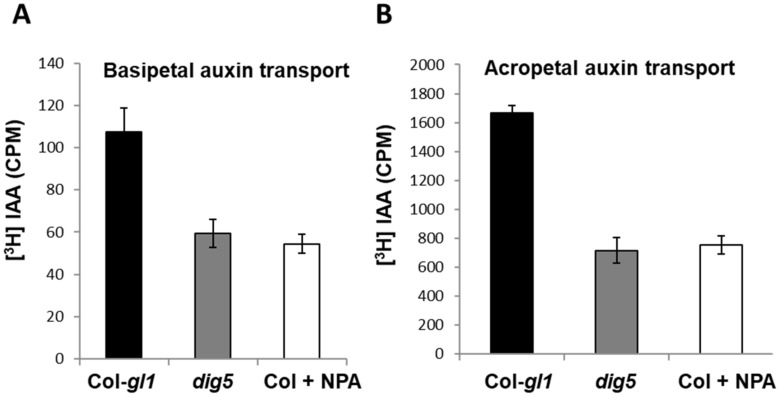
Polar auxin transport in roots of *dig5* and wild type seedlings. (**A**) Basipetal auxin transport. (**B**) Acropetal auxin transport. 100 nM [^3^H]-IAA with or without NPA (100 µM) was applied either to the root tip (**A**) or shoot-root junction (**B**) of seedlings on agar plates followed by incubation in the dark for 5 h (**A**) or 16 h (**B**). The distal segments of the roots were harvested to measure the radioactivity. Data were means and standard errors of 3 replicates, each with at least 10 seedlings.

The above data all pointed to the notion that the *dig5* mutant is likely impaired in polar auxin transport. To investigate the auxin transport capability in the *dig5* mutant, we sought to measure the basipetal (toward the base) and acropetal (toward root tip) auxin transport in *dig5* roots. We applied [^3^H]-labelled IAA to roots at either the root base or root tips and then measured the radioactivity at distal segments of the roots after a certain period of time using a method similar to that described [31]. Briefly, agar blocks containing 100 nM [^3^H] IAA with or without the auxin transport inhibitor NPA (100 μM) were applied to the root tips or root-shoot junctions, respectively. After incubation for 5 h (for basipetal) or 16 h (for acropetal) in the dark, the root samples were collected and the amount of radioactivity from the basipetal or acropetal transported [^3^H] IAA was determined using a liquid scintillation counter. As shown in Figure 5, both basipetal and acropetal IAA transport abilities in the *dig5* mutant were significantly reduced compared to those in the wild type, and the transport rate in the mutant was similar to that of the NPA-treated wild type. This indicated that *dig5* mutants were defective in both basipetal and acropetal auxin transport.

### 2.5. Map-Based Cloning of the DIG5 Locus 

**Figure 6:**
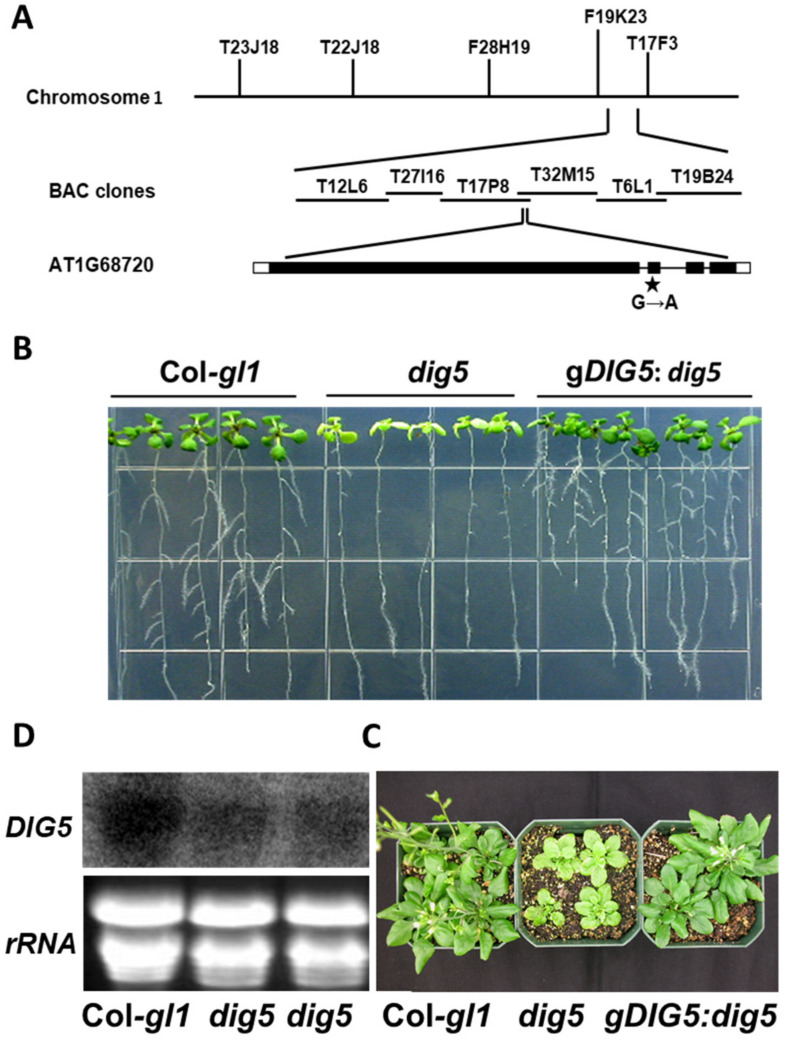
Map-based cloning of the *DIG5* locus and complementation of the *dig5* mutants. (**A**) Map-based cloning of the *DIG5* locus. After delimiting the *DIG5* locus to an interval of about 105 kb, DNA sequencing identified a single nucleotide mutation in the gene *At1g68720*. (**B**,**C**) Complementation of the *dig5* mutants with the wild type genomic DNA of *At1g68720*. Shown are the wild type Col-*gl1*, *dig5*, and *dig5* mutant transformed with the wild type *DIG5* genomic DNA (*gDIG5:dig5*) on agar plates (**B**) or in soil (**C**). (**D**) Expression level of the *DIG5* gene in the wild type and *dig5* mutant seedlings. Shown are RNA blotting of total RNA probed with the radiolabeled *DIG5* probe (upper panel) and loading control of ethidium bromide stained rRNA (lower panel).

To determine the molecular nature the *DIG5* locus, a map-based cloning strategy mapped the *dig5* mutation to the lower arm of Chromosome I (Figure 6A), within an interval of about 105 kb. All the annotated genes in the mapped interval were PCR-amplified from the genomic DNA of the *dig5* mutant and sequenced. This resulted in the identification of a single mutation in the interval. The G to A mutation at nucleotide 3511 from the predicted translational start site of the gene At1g68720 occurred at the junction between the first intron and the second exon. This mutation would alter the predicted splicing acceptor signal AG to AA and would thus shift the splicing acceptor to the immediately adjacent AG dinucleotide. Adoption of this new splicing acceptor site would result in removing this AG dinucleotide from the cDNA. RT-PCR amplification of the cDNA from *dig5* and Col-*gl1* and DNA sequencing confirmed this prediction. As a result, the mutation caused a frameshift and would produce 4 new amino acids before the transcript encountered a new pre-mature stop codon and terminate the open reading frame. RNA blotting of total RNA extracted from the wild type and *dig5* showed that the transcript level of this gene in *dig5* was lower than in the wild type (Figure 6D). This is likely caused by increased degradation of the pre-mature stop codon-containing mutant transcripts that trigger non-sense mediated decay (NMD).

To determine whether the phenotypes observed in *dig5* were caused by the mutation in *At1g68720*, we made a construct consisting of the wild type *At1g68720* genomic DNA sequence including its promoter and transferred the construct into the *dig5* mutant via the *Agrobacterium*-mediated floral dip method [32]. Multiple independent stable transgenic plants were generated and tested for their phenotypes. It was found that the *At1g68720* gene was able to rescue the leaf and the lateral root defects of the *dig5* mutants on agar media (Figure 6B) and the yellowish, short stature and other phenotypes of *dig5* mutants when grown in soil (Figure 6C). These data indicate that *At1g68720* is the *DIG5* gene and that the *dig5* mutation was responsible for the mutant phenotypes observed. 

**Figure 7:**
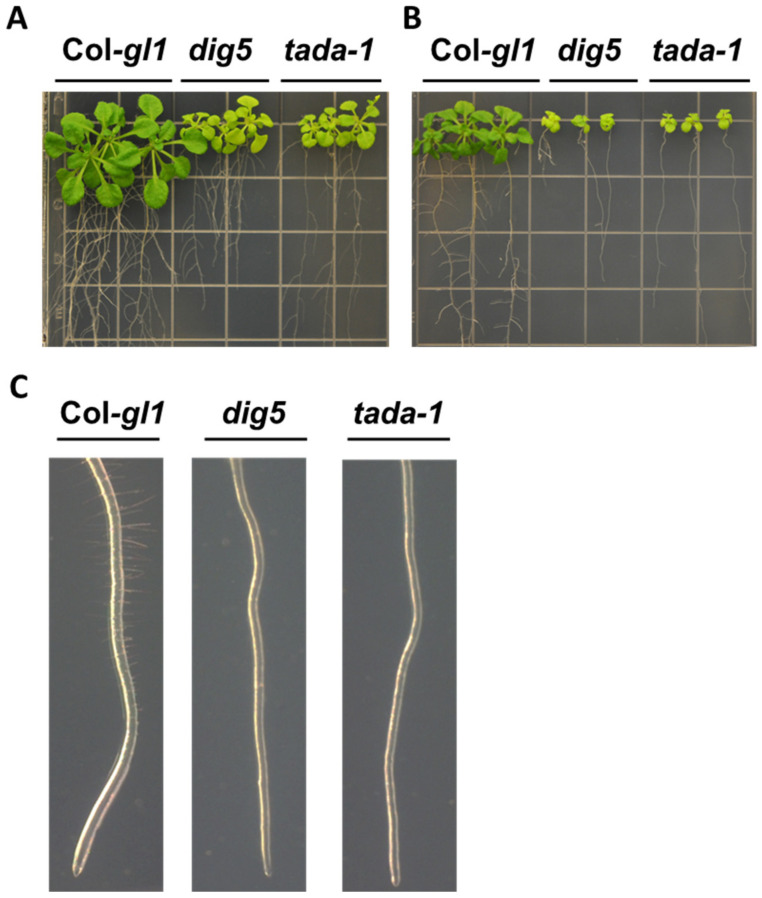
The mutant lines *dig5* and *tada-*1 had similar phenotypes. (**A**,**B**) Morphology of *dig5* and *tada-*1 seedlings on 1/2 MS without (**A**) or with (**B**) 1.0 µM ABA. (**C**) Reduced numbers of root hairs of 10-day-old *dig5* and *tada*-*1* mutant roots compared with the wild type root.

At the time we identified At1g68720 as *DIG5*, this gene was annotated as an unknown protein. The predicted protein contained a long N-terminus that showed no obvious sequence homology to other proteins, yet the C-terminus contained a short catalytic domain found in cytidine/deoxycytidylate deaminases that may participate in RNA editing in chloroplasts. Using the recombinant full-length protein or the truncated protein retaining the C-terminal catalytic domain, we failed in our attempt to detect cytosine deaminase activity. We also sequenced known RNA editing sites in related chloroplast and mitochondrial genes. However, no change in RNA editing in the mutant was detected. This gene was later reported to encode a tRNA adenosine deaminase arginine (TADA) that edited the adenosine at the wobble position of chloroplast tRNA^Arg^(ACG) to inosine tRNA^Arg^(ICG), and the mutant plants were found to accumulate lower levels of certain chloroplast proteins and to have reduced photosynthetic functions [33,34]. Phenotypes of yellowish leaves and a short stature similar to those of *dig5* were also noted with the T-DNA knockout mutant *tada* [34]. After identifying the *DIG5* locus, we obtained several T-DNA insertional alleles and an allele GK-119G08 (renamed as *tada*-1 for consistency) was used to compare their phenotypes with those of *dig5.* It was found that the *tada* mutant had phenotypes similar to those of *dig5*, such as reduced lateral root numbers (Figure 7A) and increased inhibition of primary roots by ABA (Supplementary Figure S1A,B). An additional phenotype was that both *dig5* and *tada-1* had significantly fewer root hairs than the wild type (Figure 7B,C). It is known that root hair development is also partly regulated by auxin transport [35]. Interestingly, the primary root of *tada*-*1* was less inhibited by NaCl compared with *dig5* (Supplementary Figure S1C), which likely was due to a possible leaky nature of the *tada-1* mutation since the T-DNA was inserted at the N-terminus and might have spared some of transcripts with the intact C-terminal catalytic domain to produce a low level of the functional protein. 

### 2.6. Increased Flavonoid Contents in dig5 Roots

**Figure 8:**
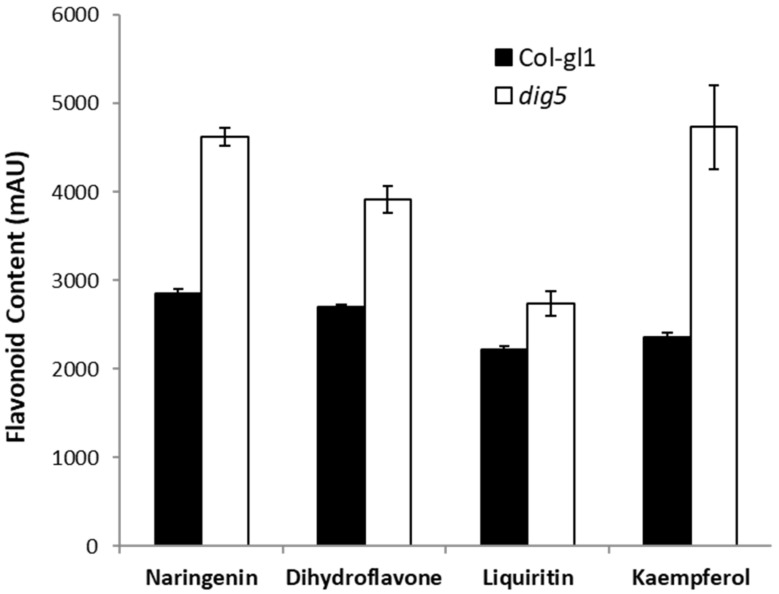
Flavonoid levels in *dig5* roots. Flavonoids in roots of 2-week-old *dig5* and wild type Col-*gl1* seedlings were extracted and quantified with HPLC (mAU, milli Absorbance Unit).

Our study showed that the *dig5* mutant was defective in polar auxin transport. Since *DIG5* did not encode a transporter but rather a putative enzyme (which we thought at the time we conducted the experiments), we hypothesized that there might be metabolite changes in *dig5* that affect auxin transport. Plants have endogenous metabolites such as flavonoids that can regulate auxin transport under physiological conditions [36]. Indeed, flavonoids, such as quercetin, kaempherol, and apigenin, are endogenous inhibitors of polar auxin transport that can regulate auxin transport and impact root development [37,38]. We therefore examined whether there were any changes in the levels of these flavonoids in *dig5*. Roots of *dig5* and wild type seedlings were harvested and extracted to measure flavonoids using an HPLC method. Our data showed that the levels of flavonoids, including naringenin, kaempherol, dihydroflavone, and liquiritin were all higher in *dig5* than in the wild type. Particularly, the level of kaempherol in *dig5* was twice that of the wild type (Figure 8). 

### 2.7. The Expression of Flavonoid Biosynthetic Genes Was Enhanced in dig5

**Figure 9:**
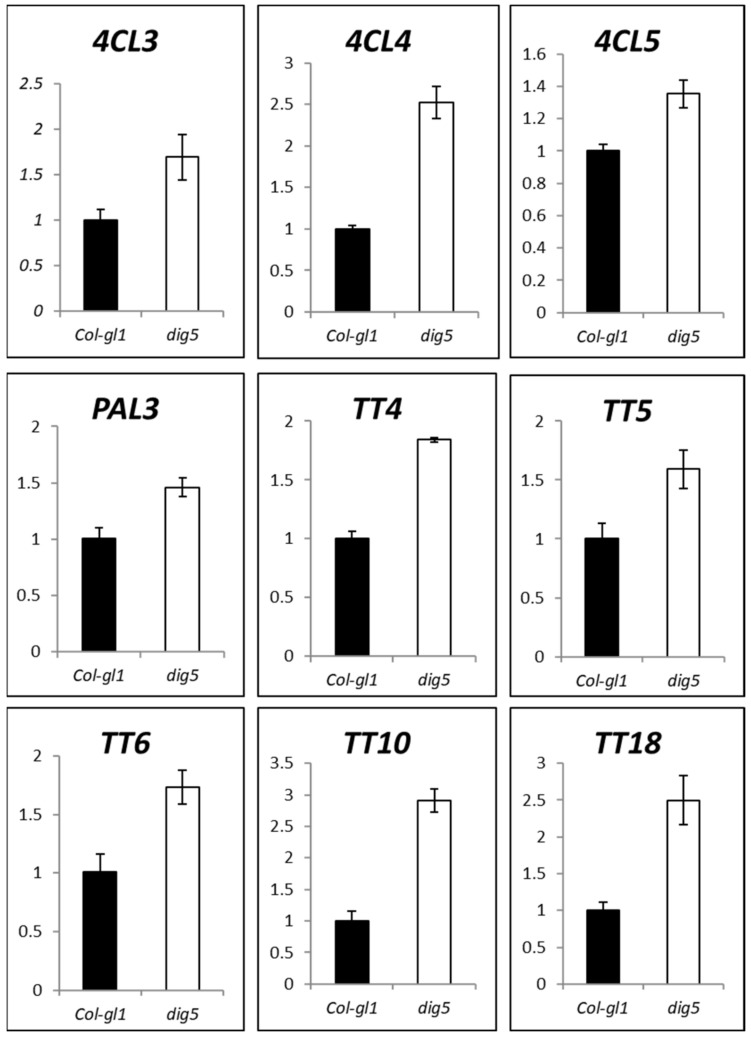
Expression of flavonoid biosynthetic genes in *dig5*. Shown are quantitative real-time PCR detection of transcript levels in roots of 2-week-old *dig5* and wild type (Col-*gl1*) seedlings. *4CL3/4CL4/4CL5* encode 4-coumarate:CoA ligase 3/4/5; *PAL3* encodes phenylalanine ammonia-lyase 3; *TT4* encodes chalcone synthase (CHS); *TT5(At3g55120)* encodes chalcone isomerase (CHI); *TT6* (*At3g51240)* encodes flavanone 3-hydroxylase; *TT10 (At5g48100)* encodes a laccase-like polyphenol oxidase; *TT18 (At4g22880)* encodes anthocyanidin synthase (ANS).

The fact that *dig5* mutants had higher contents of flavonoids suggests that there may be increased biosynthesis of these metabolites. To test this possibility, we performed quantitative real-time PCR to measure the expression of major flavonoid biosynthesis genes in the roots of *dig5* in comparison with the wild type. The flavonoid biosynthesis pathways are well studied in Arabidopsis and other plants, through genetic and biochemistry approaches [39,40,41]. Flavonoids are synthesized from phenylalanine through the phenylpropanoid pathway where the first enzyme of the pathway phenylalanine ammonia-lyase (PAL) converts phenylalanine into cinnamate, which is then converted to ρ-coumarate. ρ-coumarate is further catalyzed by 4-coumarate coenzyme A ligase (4CL) to form 4-coumarate coenzyme A (CoA). Chalcone synthase (CHS/TT4) then combines 4-coumarate CoA with malonyl-CoA to produce naringenin chalcones. Chalcone isomerase (CHI/TT5) isomerizes chalcones into naringenin, which is catalyzed by flavanone 3-hydroxylase (F3H/TT6) into dihydroflavones. In the late part of the pathway, flavanol synthase (FLS) converts various dihydrofavones into common flavanols, such as kaempferol and quercetin [40]. Our quantitative real-time PCR analyses found that there was significantly increased expression of major genes in the flavonoid biosynthesis pathway: *PAL3*, *4CL3*, *4CL4*, *4CL5, TT4*, *TT5*, *TT6*, *TT10*, and *TT18* (Figure 9). The higher expression of these genes in *dig5* may contribute to increased syntheses of flavonoids in the mutant.

### 2.8. Genetically Blocking Flavonoid Synthesis Rescued dig5 Phenotypes

**Figure 10:**
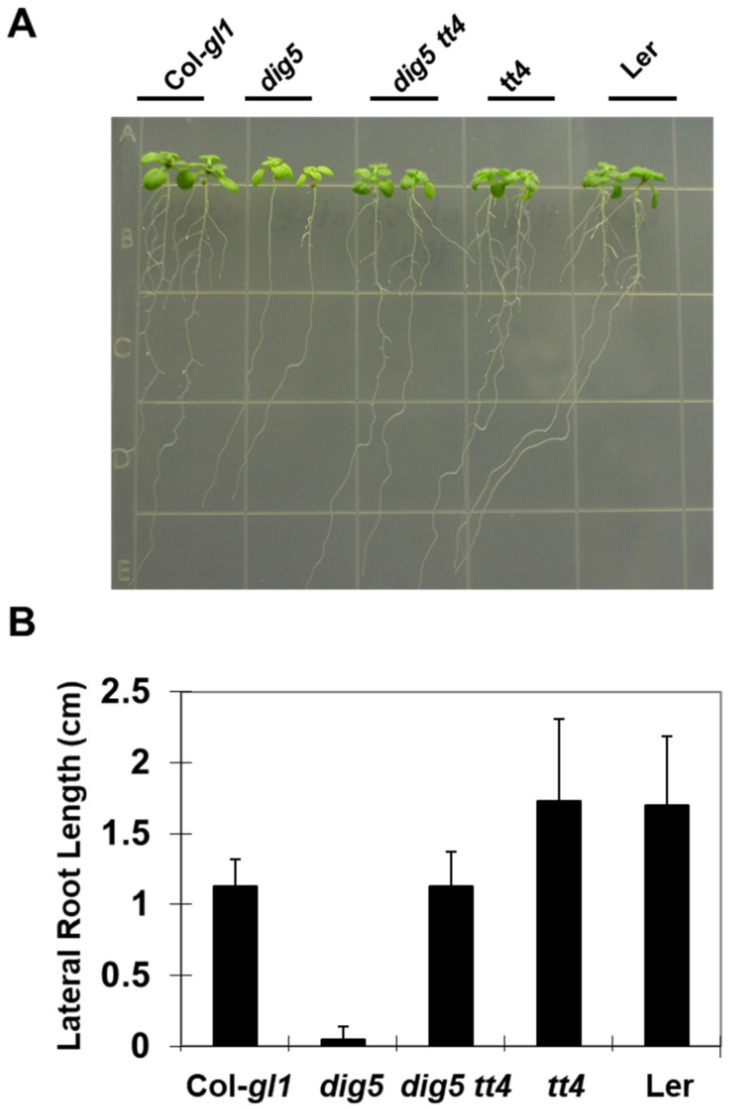
Loss-of-function mutation of *TT4* rescued the development defects in lateral roots of *dig5* mutant seedlings. The *dig5 tt4* double mutant was generated by genetic crossing. (**A**) Morphology of 2-week-old seedlings grown on 1/2 MS medium. (**B**) Total lateral root length per plants. Data are means and standard deviation from 6 seedlings.

In the *dig5* mutant, the enhanced expression of flavonoid biosynthetic genes and the higher levels of flavonoids in roots of *dig5* are consistent with the impaired auxin transport and the reduced lateral root development in the mutant. To determine whether the increased flavonoid content was the cause of these phenotypes, we made a double mutant between *dig5* and *transparent testa 4* (the *tt4-1* allele*)* by crossing the single mutants. *TT4* encodes a chalcone synthase, which catalyzes the first committed step in the flavonoid pathway. The *tt4* mutant has very low levels of all major flavonoids [37,41,42]. The *dig5 tt4* double mutant along with the single mutants and Col-*gl1* (background of *dig5*) and Landsberg-*erecta* (*Ler)* (background of *tt4*) were germinated and one-week-old seedlings were transferred to MS medium to grow for 10 days (Figure 10A,B). While *dig5* had no or very few lateral roots, the *dig5 tt4* double mutant had a lateral root phenotype close to that of the wild type Col-*gl1*. Measurement of the total lateral root length per seedling showed that *tt4* suppressed the *dig5* lateral root phenotype, suggesting that *TT4* acts downstream of *DIG5* in controlling lateral root development and that higher levels of flavonoids may underlie the lateral root defects in the *dig5* mutant.

## 3. Discussion

The plant root system is of critical importance for plants fighting drought stress. Roots not only mine water from soil, but can also coordinate other processes of the whole plant to manage the water economy. Complicated mechanisms are expected to exist in plants that regulate these processes, yet few of these mechanisms are well elucidated. A consensus is that a robust root system that can forage water from deeper soil (also known as a drought avoidance strategy) would enhance plant tolerance to drought stress. In this regard, allocation of limited resources to grow deeper roots will be of adaptative advantage to the plants. Reduced lateral root growth of Arabidopsis in response to osmotic stress and ABA is thus considered to be one of such adaptative strategy [25,43]. This altered growth habit underlies an important aspect of the plasticity of root developmental adaptation to the environment. Although the exact mechanisms for this adaptive response are not very clear, the involvement of phytohormones, particularly auxin and ABA, in the process has been implicated. The identification of the *DIG5* locus in this study highlights further an unexpected route of regulation of lateral root development. 

Auxin control of lateral root development has been well documented [20,21,44,45]. Nonetheless, the interaction between auxin and drought or ABA in regulating the root response to drought is less well understood. Drought stress may alter auxin biosynthesis, catabolism, transport, and signaling to affect root development. For instance, drought or ABA enhances the production of the microRNA miR393, which targets the transcripts of auxin receptor genes, such as *TIR1* and *AFB2,* for cleavage and degradation. In transgenic plants expressing miR393-resistant versions of *TIR1* or *AFB2*, these mutant transcripts are no longer cleaved and neither ABA nor osmotic stress (generated by polyethylene glycol) inhibits the elongation of lateral roots [46]. Thus, auxin signaling is important for regulating lateral root growth during plant response to drought stress.

Although auxin can be synthesized locally, long distance polar auxin transport is important for organogenesis including lateral root development [45]. Several classes of auxin transporters are described, among which the PIN family of auxin efflux carriers are of particular importance and have been extensively investigated [47]. The functionality of these carriers is determined by their transport activity and polar localization, both of which are regulated by various processes such as phosphorylation [48]. Besides these posttranslational modifications, cellular metabolites appear to regulate the transporter activities as well. Among these metabolites, flavonoids have been widely reported to inhibit auxin transport [36].

Flavonoids are synthesized through the phenylpropanoid pathway. The first committed step in the flavonoid pathway is catalyzed by chalcone synthase (CHS) encoded by the *TT4* (*transparent testa 4*) gene in Arabidopsis. The other major steps of the pathway have also been defined genetically with other *tt* mutants, such as *tt5*, *tt6*, *tt7,* etc. [40,41]. Mutations in these genes can significantly decrease flavonoid accumulation. In *dig5* mutant roots, levels of several flavonoids increased significantly, particularly kaempferol, which is a major flavonoid that accumulates in Arabidopsis roots [40,49]. This increased flavonoid accumulation was likely caused by the increased expression of flavonoid biosynthetic genes. Indeed, the expression of major genes in the early steps in the pathway increased significantly (Figure 8). Another possibility is that more substrates of the pathway may have channeled into flavonoid production. 

Flavonoids have important functions in processes, such as UV tolerance, oxidative stress response, defense response, and abiotic stress, as well as bacterium–host interactions [38,50,51,52]. The involvement of flavonoids in regulating auxin transport has long been known [36,38,42], and the discovery of synthetic auxin transport inhibitors such as NPA that mimic flavonoids makes chemical regulation of auxin transport much easier. Several targets of NPA or flavonoids in auxin transport have been proposed [53]. Recent studies suggested that the PIN family of polar auxin efflux carriers are the major targets. Flavanols and NPA stabilize PIN dimers, thus inhibiting the transport activity of these auxin efflux carriers [54,55]. Recent structure analyses shed light on how NPA may affect auxin transport, e.g., by competing for the IAA-binding site of PIN transporters [56,57]. In *dig5*, multiple phenotypes indicative of auxin transport defects were observed. These include reduced lateral root growth, abnormal root curling, loose apical hook formation, and reduced number of root hairs, etc. Measurement of auxin polar transport in roots indeed showed that both acropetal auxin transport and basipetal auxin transport were significantly reduced in the *dig5* mutant (Figure 8). We therefore suggest that increased accumulation of flavonoids in *dig5* plants impairs auxin transport, which in turn leads to reduced lateral root growth and the other phenotypes related to defects in auxin transport. Consistently, introduction of the *tt4* mutation (which abolishes flavonoid production) into the *dig5* mutant restored the lateral root growth to the level of the wild type (Figure 10), indicating an important role of DIG5 in maintaining flavonoid homeostasis in plants.

Our map-based cloning uncovered that *DIG5* encodes a protein that was subsequently identified as tRNA adenosine deaminase Arginine (TADA) [34]. TADA catalyzes the deamination of the adenosine at the wobble position of tRNA^Arg^ (ACG) to inosine in tRNA^Arg^ (ICG) in chloroplasts/plastids. The *tada* mutation reduced chloroplast translation efficiency and led to reduced levels of certain plastid-encoded proteins and impaired photosynthesis functions [34,58]. These affected proteins, however, are not directly involved in the phenylpropanoid biosynthesis pathway. Thus, the defect in plastid protein homeostasis may generate signal(s) that affect the expression of nuclear genes involved in flavonoid biosynthesis. Retrograde signaling from chloroplasts or mitochondria has been shown to be common in regulating nuclear gene expression [59]. Mutants of several chloroplast genes were reported to affect lateral root development. For instance, the Arabidopsis mutants of the chloroplast-localized protein FIERY1 (FRY1) [60] had fewer lateral roots and were more sensitive to ABA inhibition of lateral root growth [61,62]. The plastidial glycolate and glycerate transporter mutant *plgg1* [63] and the NADPH thioredoxin reductase C mutant [64] also had reduced lateral roots. That chloroplast retrograde signaling inhibits lateral root development is likely of adaptive advantage to the plant. Drought stress is known to cause chlorophyll breakdown and leaf senescence, which, as shown in the current study, may trigger the production of flavonoids to inhibit horizontal root growth and to promote roots to grow deeper to increase drought tolerance of the plant. This possibility can be tested in future studies.

How chloroplast defects may trigger flavonoid production and inhibit lateral root development is not yet clear. In the current study, we speculate that the defects in chloroplast translation caused by lack of DIG5/TADA protein may lead to an imbalance in the chloroplast of, e.g., reactive oxygen species, which in turn activates the expression of flavonoid genes in the nucleus in order to maintain organellar redox homeostasis in the *dig5* mutant. The increased accumulation of flavonoids would result in reduced auxin transport and impaired lateral root development. The exact mechanisms through which chloroplast protein homeostasis triggers the retrograde signaling to activate nuclear flavonoid biosynthetic genes warrant further investigation. 

## 4. Materials and Methods

### 4.1. Plant Materials and Growth Conditions

Arabidopsis (*Arabidopsis thaliana*) ecotype Col-0 carrying the *glabrous1(gl1)* mutation (referred to as Col-*gl1* or the wild type) was used to conduct mutagenesis with ethyl methanesulfonate. Mutant screening of the M_2_ generation for altered lateral root growth responses under treatment of mannitol or ABA was described previously [25]. Isolated mutants were re-screened and backcrossed to Col-*gl* twice before being used for further characterization. For general growth assays, unless otherwise stated, seeds were surface sterilized and planted on 1/2 MS media (1.2% agar and 3% Sucrose). The plates were then incubated at 4 °C for 3 d before being placed vertically under constant white light at 22 °C for germination and seedling growth. 

### 4.2. Root Growth, Hypocotyl Elongation, Apical Hook Formation and Auxin Responses 

For root growth assays, 5-day-old seedlings were individually transferred from 1/2 MS medium plates with a pair of forceps to the treatment medium plates and incubated in an incubator for a specified period before taking photographs or measurements.

For root curling assay, the surface-sterilized seeds were sown on 1/2 × MS medium agar plates without or with 5 μM NPA supplement. After cold treatment, plates were incubated for 2 days in the light and then 5 days in the dark (all at 22 °C) before taking pictures from the back of the plates. For hypocotyl elongation measurements, seedlings were germinated and grown on the surface of the medium in a vertical position for 5 days at 22 °C in the dark. For apical hypocotyl hook observation, plates were wrapped with aluminum foil and incubated at 22 °C in a vertical position for 3 days before taking pictures. For the hook formation assays, NPA was used at 1 µM and ACC was at 10 µM. 

To test the auxin responses, IAA, NAA or 2,4-D was added into the medium separately with the final concentration at 100 nM, 10 nM, and 1 nM, respectively. The phenotypes of roots were observed, and the pictures were taken with a digital camera. 

Length of lateral roots and primary roots were measured with the digital images of seedlings using the Image J software as described [25]. Number of visible lateral roots per seedling was counted and the density was calculated by dividing with the length of the primary root.

### 4.3. Chlorophyll a and b and Carotenoid Measurements

For pigment measurements, fresh leaves of 3-week-old seedlings grown in soil were ground in absolute ethanol. After centrifugation, the supernatant was used to measure the absorbance at 663, 645, and 480 nm. Pigment contents were calculated as described [65].

### 4.4. Leaf Venation Observation

Fresh leaf tissues were fixed in saturated chloral hydrate. The samples were then mounted on glass slides with coverslips, the leaf veins were visualized, and the pictures were taken with differential interference contrast (DIC) images using a Nikon SMZ1500 microscope and a Qimaging Retiga cooled camera (Burnaby, BC, Canada).

### 4.5. Auxin Transport Assays

The basipetal and acropetal auxin transport capabilities of seedlings were measured according to the method described [31], with modifications. The growth medium contained 0.8% agar, 1 × MS, and 1.5% sucrose, at pH 5.7. Seeds were germinated, and seedlings were grown on vertically placed agar petri dishes for about 5 days until the roots reached the length of 1.0–1.5 cm. Seedlings were transferred to treatment plates with root tips aligned. ^3^H-IAA (American Radiolabeled Chemical, St. Louis, MO, USA) at 100 nM was mixed with 1% (*v*/*v*) agar either with or without 100 μM NPA and was applied to localized areas of roots for transport assays. For basipetal auxin transport, the radiolabeled IAA-containing agar was applied to just touch the root tip. Plates remained vertically oriented for 5 h in the dark to minimize IAA breakdown. For acropetal transport assay, 10 μM cold IAA was added as it could increase ^3^H-IAA transport. The ^3^H-IAA agar block was placed at the root-shoot junction of the seedlings. Plates were incubated in the dark for 16 h with seedlings upside-down. Individual 2- or 5-mm segments were cut and rinsed and placed into 2.5 mL of scintillation fluid. The radioactivity was measured using a liquid scintillation counter. Three independent experiments were conducted, each with at least 10 individual seedlings. 

### 4.6. HPLC Detection of the Flavonoid Content in Roots

Roots of seedlings vertically growing on agar petri dishes for 2 weeks were harvested and grounded immediately in liquid N_2_. The samples were then extracted with 80% (*v*/*v*) methanol at 1 g/10 mL at room temperature. After centrifugation (4000× *g*, 5 min) and filtration (0.45 μm syringe filter), the samples were re-extracted with ethyl acetate. Extracts were concentrated to dryness with an Eppendorf vacufuge at 45 °C and dissolved in 80% methanol for HPLC analysis. The extracts were analyzed on an Agilent 1100 series HPLC system (Agilent, Santa Clara, CA, USA) using authentic standards as internal controls as described [66].

### 4.7. Constructs and Arabidopsis Transformation

The full-length cDNA of *DIG5* was amplified and cloned into the *Ecor I* and *Sal I* sites of the *pZP* vector. The genomic sequence of *At1g68720* was also amplified from Col-0 plants and used in the complementation assay. The constructs were introduced into *Agrobacterium tumefaciens* strain GV3101 and then transformed into the *dig5* mutant using the floral dip method [32]. The transgenic plants were screened on a MS agar medium containing 50 mg/L kanamycin.

### 4.8. Quantitative Real-Time PCR

Total RNAs were extracted from 10-day-old seedlings using the RNeasy Plant Mini kit (Qiagen), and RT reactions were conducted with SuperScript III First-Strand Synthesis SuperMix (Invitrogen) using random hexamers for the first strand synthesis. After 10 times dilution, 1 µL of the diluted solution was used as a template in a 10-µL reaction with 2 × SYBR Green SuperMix, ROX (Invitrogen). The quantitative RT-PCRs were performed in triplicate using the ABI 7900HT Fast Real-Time PCR System (Applied Biosystems). *UBQ3* was used as internal control. Primer sequences are listed in Supplementary Table S2.

## Data Availability

All data generated or analyzed during this study are included in this published article.

## Supplementary Materials

The following supporting information can be downloaded at: https://www.mdpi.com/article/10.3390/ijms231810642/s1.
