# Supplementary material for: The Plastidial DIG5 Protein Affects Lateral Root Development by Regulating Flavonoid Biosynthesis and Auxin Transport in Arabidopsis
Source: Int J Mol Sci. 2022 Sep 13;23(18):10642. doi: 10.3390/ijms231810642 (PMC9501241; doi:10.3390/ijms231810642)
Supplement: Supplementary file 1 [file ijms-23-10642-s001.zip › ijms-1891279-supplementary.pdf]

Supplementary Materials

Supplementary Table S1. Morphometric Analysis of the Wild Type and *dig5* mutant

Measurements	Wild Type	*dig5*
Length of the primary root after 8 days	6.3±0.6	3.7±0.5
Number of lateral roots after 8 days	8.5±0.6	1.4±0.2
Height of main inflorescence (cm)	21.7±0.9	6.2±1.5
Number of siliques on primary inflorescences	7.1±0.7	1.9±0.4
Distance between siliques (mm)	12.9±0.8	6.9±0.8

[a] Data are means and SEs of eight plants

Supplementary Table S2. Primers used in this study

Table S2 Primiers used in this study

Primer names	Sequences (5' to 3')
Primers for full length cDNA cloning	
225cdna-F	CACCATGTTCAATACATACAC
225cdna-R	CAGACAGAAAAAGGGTAAGACTAG
Primers for promoter cloning	
225P-F	CACCCCTATTGTAAATTGCACACC
225P-R	GTTGAATTAGAAGAGGTACCTTG
Primers for full length genomic DNA cloning	
225G-R	GCGTACGCTACAATCAATGGC
225G-F(same with 225P-F)	CACCCCTATTGTAAATTGCACACC
Primers for overexpression	
225oe-F	CACCCAAGGTACCTCTTCTAATTC
225oe-R	CTACAGACAGAAAAAGGGTAAGAC
Primers for qPCR	
4CL1-QF	GCCGTTAATGCCAAGTTTCC
4CL1-QR	GAAAGAGAATCTCCGGTGTCTG
4CL2-QF	CTATGGGATGACAGAAGCAGG
4CL2-QR	GTATCTTCATCTCGGCGTTCC
4CL3-QF	TTCTCCTTCATGGGTGCTTC
4CL3-QR	GGTGTTGGTTCATCGGTAGTG
4CL4-QF	AGGCTTGTACTCTGTTTGGTG
4CL4-QR	CGCTGAAGGGTGTTGAAGTAG
4CL5-QF	CTCTGTCTTTTCTTGCCGTTG
4CL5-QR	GTTGTCACCGTCATCGTCTAG
4CL6-QF	TCTGATTTCCGTGAAGTGACTC
4CL6-QR	TCCTGTCTTTGGCTTGATCTG
4CL7-QF	GCTCGTCCAGTTCATAGATCAG
4CL7-QR	CAACAGCTCCACCGTTACTAG
4CL8-QF	TGGGAATACGGAGAGGTGAC
4CL8-QR	GTGTTGAGAGGATTAGCGGTAG
C4H-QF	CCGGGAAAGGTCAAGATATGG
C4H-QR	TCAAACTCCCAACCTTCACG
PAL3-QF	AGCGGTTAATGAGGTTGTGAG
PAL3-QR	TTAAAGGGTTAGTGAGGCTGC
TT1-QF	AACCCTAATCCCTTGTATGCG
TT1-QR	GCGATCTCCTTCCCATTTCTC
TT3-QF	TTCATCGGTTCATGGCTAGTG
TT3-QR	ACCGTCACATCCGTTTATGG
TT4-QF	CTTACATGGCTCCTTCTCTGG
TT4-QR	TGATCTTTGACTTGGGCTGG
TT5-QF	GCCGTTCCTTCTCTATCTGTC
TT5-QR	AATTCTCCGTCACTTTCTCCG
TT6-QF	ATCGTCTCTAGTCACCTCCAG
TT6-QR	TCACTTTCACCCAACCTTCC
TT7-QF	AAGCCTCATCGAACCCTTTC
TT7-QR	TGCCATGTGTTTAGCTCCTG
TT8-QF	GAGCTATTCTCGCTAAGAGTGC
TT8-QR	CGTAGGTGGAGTGTTCAGAAAG
TT10-QF	GTGGAAGAGGGATGTGAGAG
TT10-QR	AGCAAGGATACAAGAAACCAGG
TT18-QF	ACTATCCAAAATGTCCTCAGCC
TT18-QR	TCTCCAAAGTATCCCCAATGTG

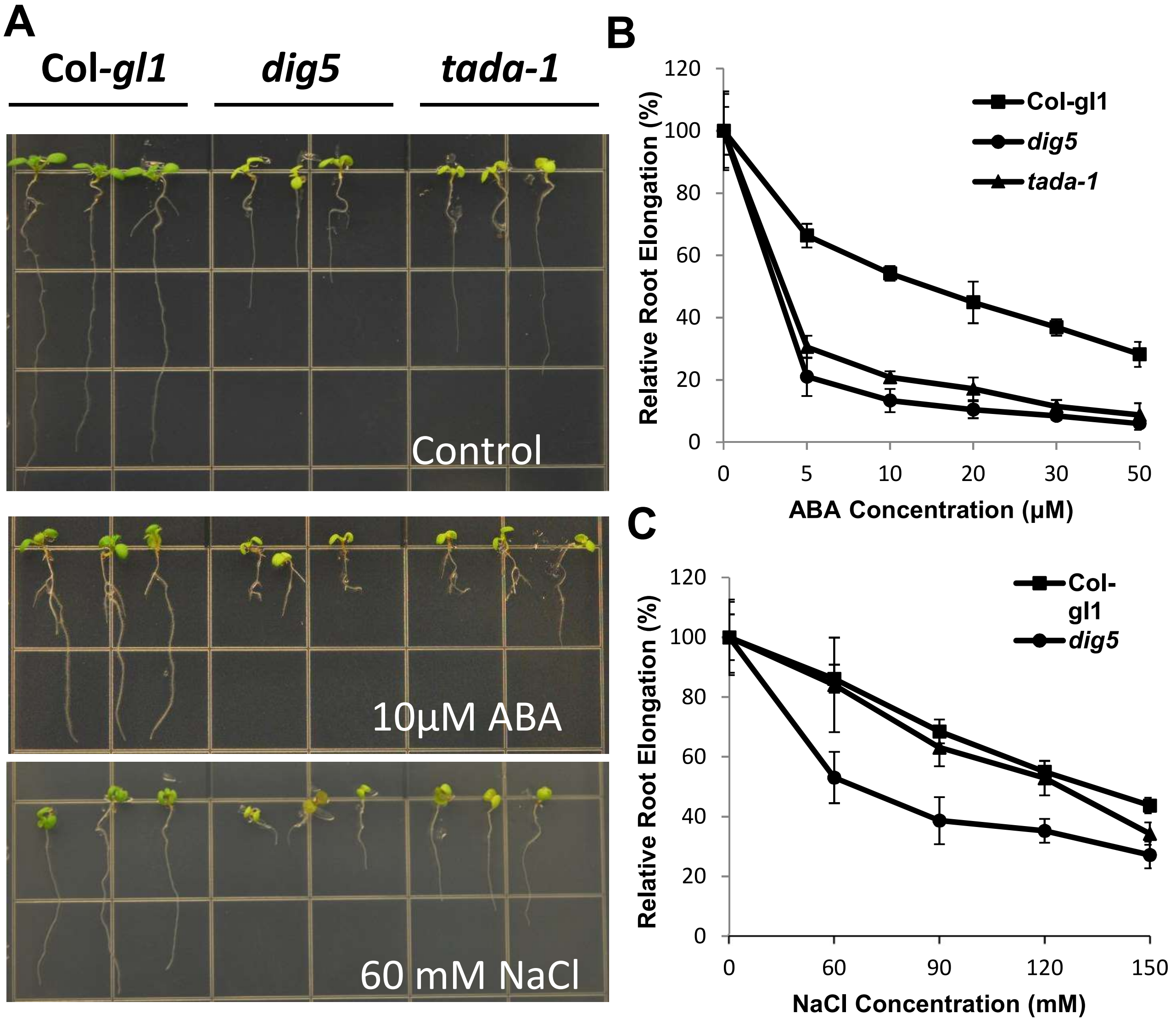

Supplementary Figure S1. Primary root growth of *dig5* and *tada-1* mutants under ABA and NaCl treatments. A. Morphology of seedlings on treatment plates. B. Relative root elongation under ABA treatments. C. Relative root elongation under NaCl treatments.
